# Supplementary material for: Analyzing dwell times with the Generalized Method of Moments
Source: PLoS One. 2019 Jan 8;14(1):e0197726. doi: 10.1371/journal.pone.0197726 (PMC6324800; doi:10.1371/journal.pone.0197726)
Supplement: S1 Supporting Information — Includes all supporting information referred to in the main text. Supporting Information contains all figures, complete results, experimental data, as well as the mathematical proof referred to in the main text. (PDF) [file pone.0197726.s001.pdf]

SUPPORTING INFORMATION

Analyzing dwell times with the Generalized Method of Moments

Sadie C. Piatt, Allen C. Price*

Emmanuel College, Department of Chemistry & Physics
400 The Fenway
Boston MA 02115

*E-mail address: priceal@emmanuel.edu

For each data point in the following figures, mean and mean deviation for 1,000 trials of the condition described are plotted. Residence time estimates are plotted as a function of sample size. Figures are displayed as panels including pairs of residence times. Row 1 is the first pair of residence times (10 s, 10 s), row 2 is the second pair (10 s, 20 s), row 3 is the third pair (10 s, 50 s), and row 4 is the fourth pair (10 s, 100 s). The estimate for the smaller time (always 10 s) is shown on the left, and the estimate for the larger time (10, 20, 50, or 100 s) is shown on the right.

S1. Diagonal jackknife weight matrix comparison

The complete results for the D-matrix weighting scheme (see Materials and Methods for full description) as applied to a two-step reaction mechanism are shown in Figure S1. For the residence times which are not equal to each other (C/D, E/F, G/H), the bias generally decreases as N increases and as order decreases. The only exception is the smaller estimate for the parameter set (10, 20 s), where there is little variation with respect to cumulant order. For the smaller estimate when the two residence time parameters are equal (A), there is also little variation with respect to cumulant order, but estimates converge to a value less than the true parameter (9 vs. 10 s). For the larger estimate under these conditions (B), second order produces positive bias and fourth order produces negative bias. Estimates here converge to a value greater than the true parameter (11 vs. 10 s).

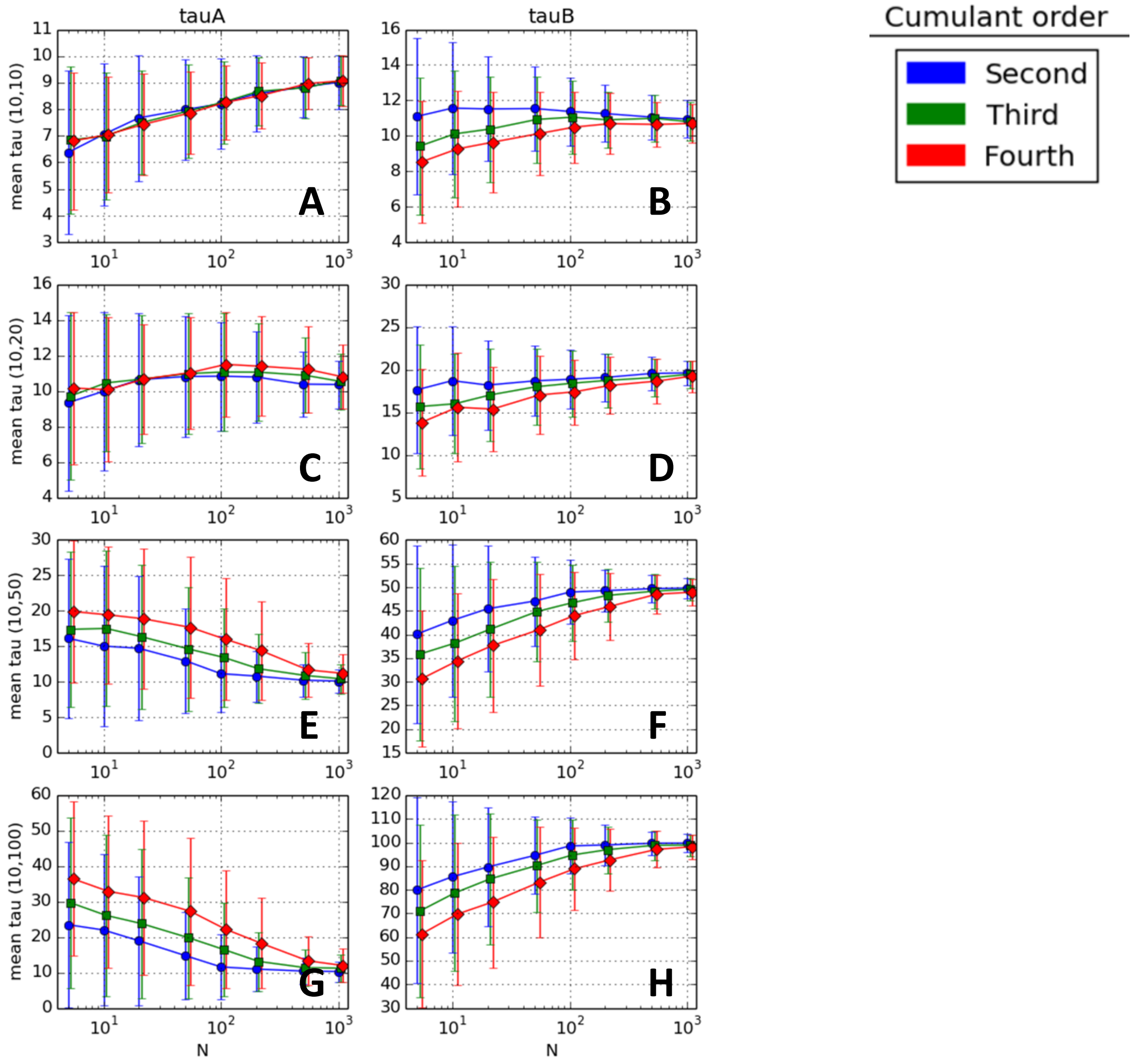

FIGURE S1. Results for D-matrix weighting.

S2. Full covariance weight matrix comparison

The complete results for the C-matrix weighting scheme (see Materials and Methods for full description) as applied to a two-step reaction mechanism are shown in Figure S2. For the larger estimate (D, F, H), the bias generally decreases as N increases and as order decreases. This is also generally true for the smaller estimate (C, E, G), but third and fourth order cumulants do not consistently increase or decrease in bias. For the smaller estimate when the two residence time parameters are equal (A), bias generally decreases as N increases and order decreases, but estimates converge to a value less than the true parameter (9 vs. 10 s). For the larger estimate under these conditions (B), second order produces positive bias and fourth order produces negative bias. Estimates here converge to a value greater than the true parameter (11 vs. 10 s).

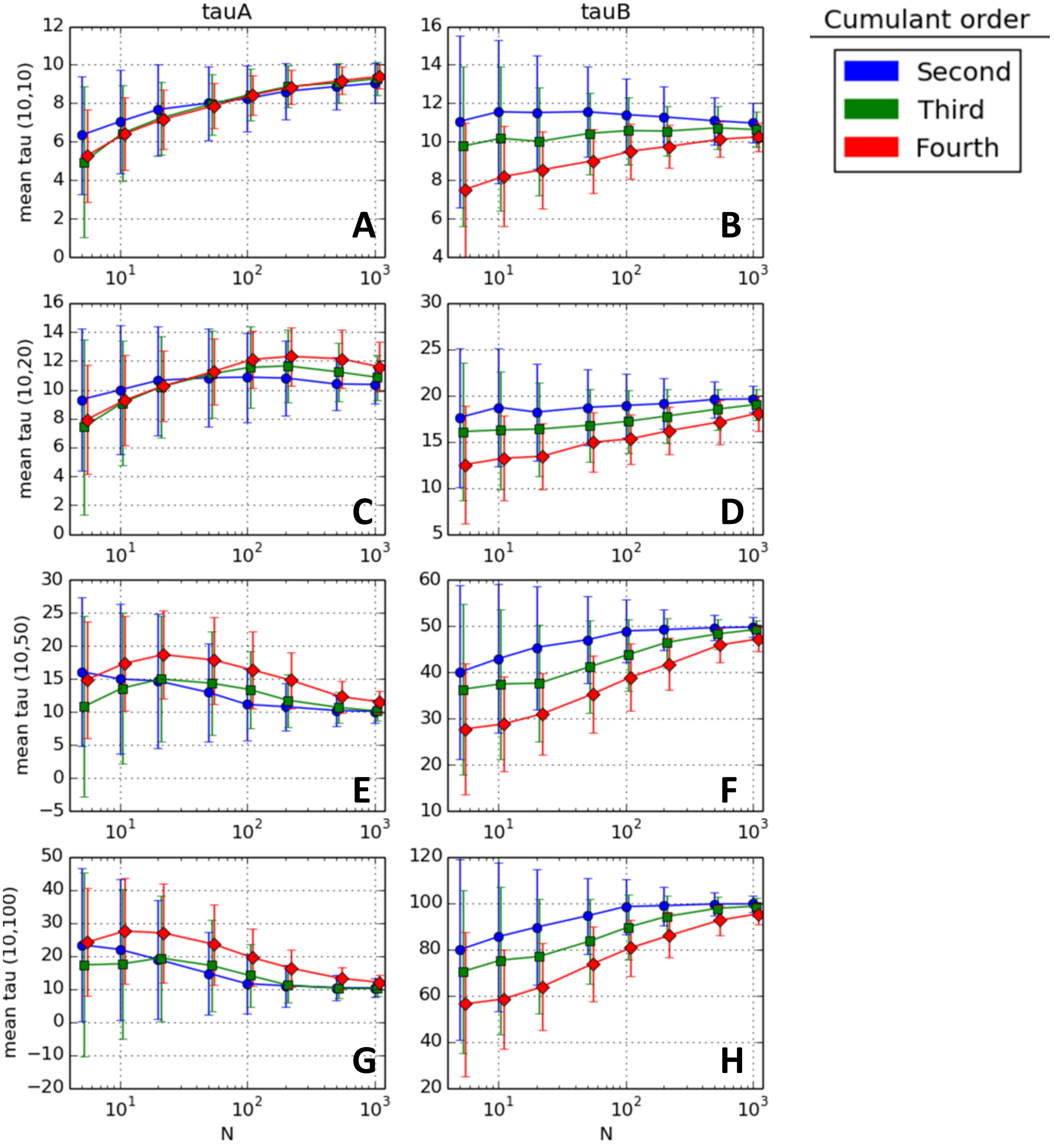

FIGURE S2. Results for C-matrix weighting.

S3. Identity weight matrix comparison

The complete results for the I-matrix weighting scheme (see Materials and Methods for full description) as applied to a two-step reaction mechanism are shown in Figure S3. For the larger estimate (B, D, F, H), the bias generally decreases as N increases and as order decreases (where values converge to 11 instead of 10 s for B). This is not generally true for the smaller estimate (A, C, E, G). For the smaller estimate when the two residence time parameters are equal (A), bias generally decreases as N increases and order decreases, but estimates converge to a value less than the true parameter (9 vs. 10 s). Additionally, the fourth order cumulant has considerable bias at low N and the least bias at high N. For the smaller estimate when the two residence time parameters are not equal (D, F, H), third and fourth order cumulants do not have consistent bias behavior with respect to N, and second order cumulants have the least bias.

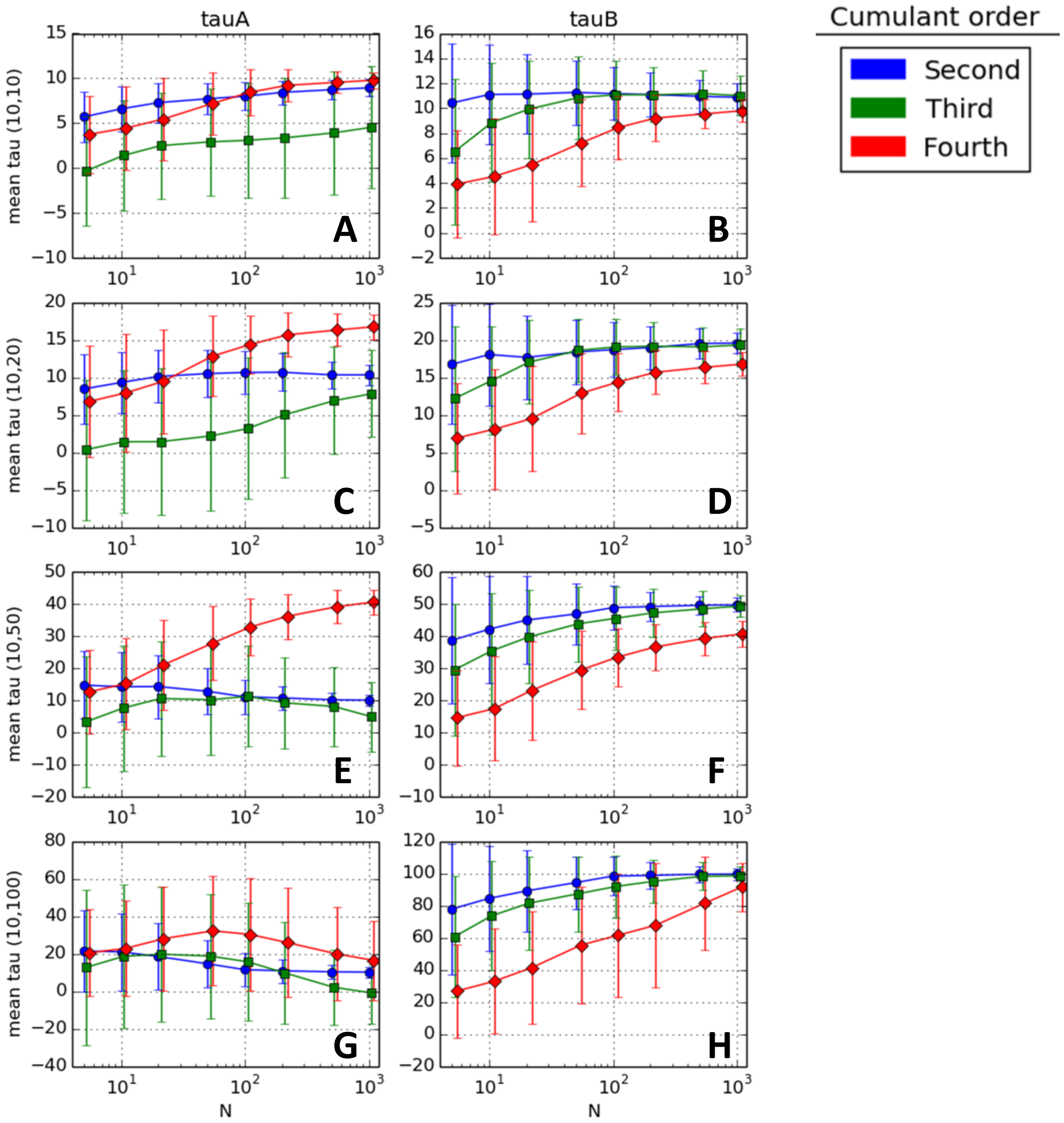

FIGURE S3. Results for the I-matrix weighting.

The following three figures (S4, S5, and S6) display the same results as figures S1, S2, and S3. However, rather than holding cost function weight matrix constant, cumulant order is held constant and all weight matrices are compared.

S4. Second-order cumulant cost function comparison

The complete results for the second order cost function (see Materials and Methods for full description) as applied to a two-step reaction mechanism are shown in Figure S4. For all estimates, the bias generally decreases as N increases. There is little variation with respect to the weight matrix, where the identity matrix is slightly more biased for all estimates. For the smaller estimate when the two residence times are equal (A), estimates converge to a value less than the true parameter (9 vs. 10 s). For the larger estimate under these conditions (B), estimates converge to a value greater than the true parameter (11 vs. 10 s).

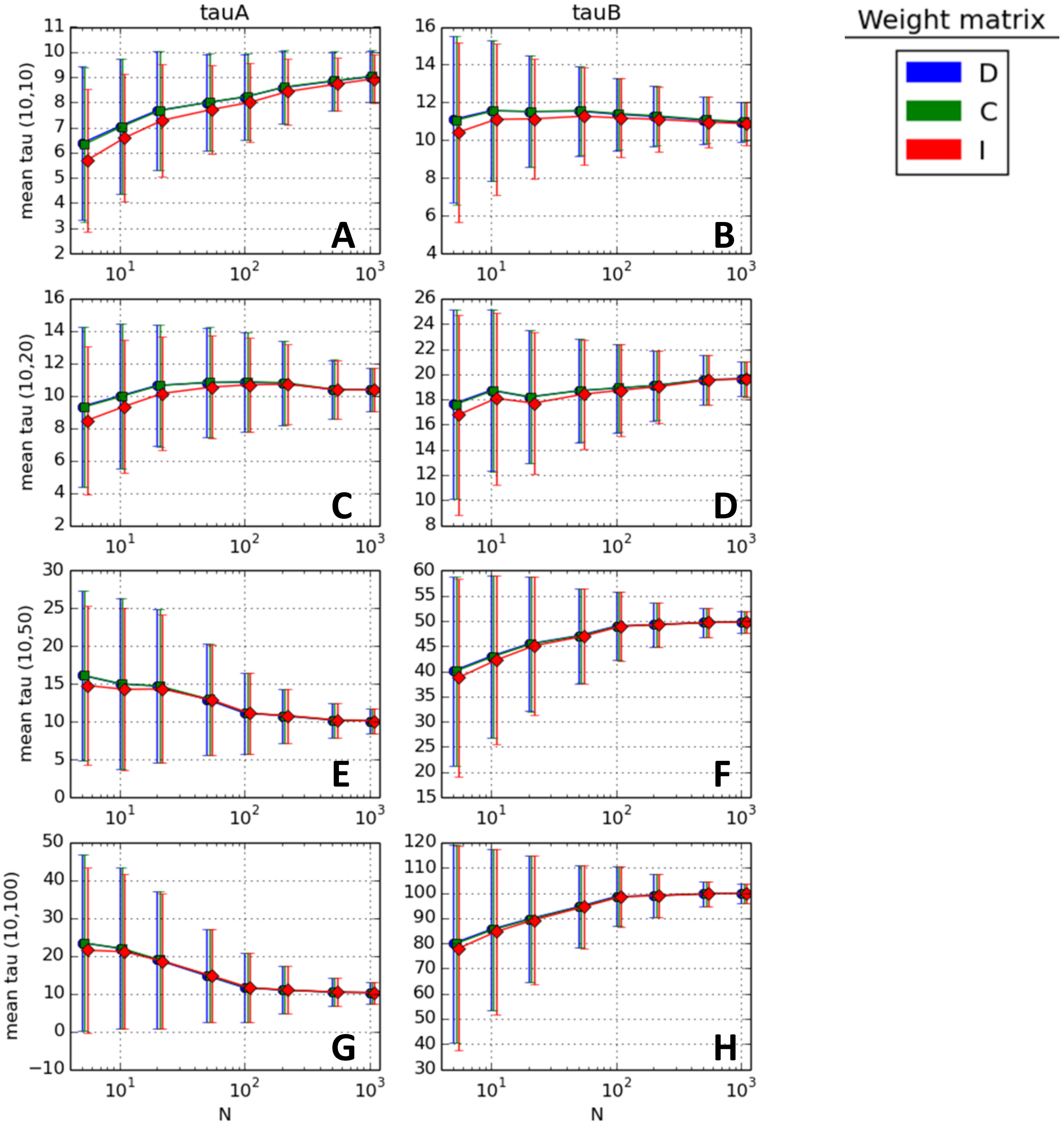

FIGURE S4. Results for second-order cost function.

S5. Third-order cumulant cost function comparison

The complete results for the third order cost function (see Materials and Methods for full description) as applied to a two-step reaction mechanism are shown in Figure S5. For all estimates, the bias generally decreases as N increases. While the diagonal jackknife weight matrix and the full jackknife matrix generally have comparable bias, the identity matrix is consistently more biased for all estimates. For the smaller estimate when the two residence times are equal (A), estimates converge to a value less than the true parameter (9 vs. 10 s). For the larger estimate under these conditions (B), estimates converge to a value greater than the true parameter (11 vs. 10 s).

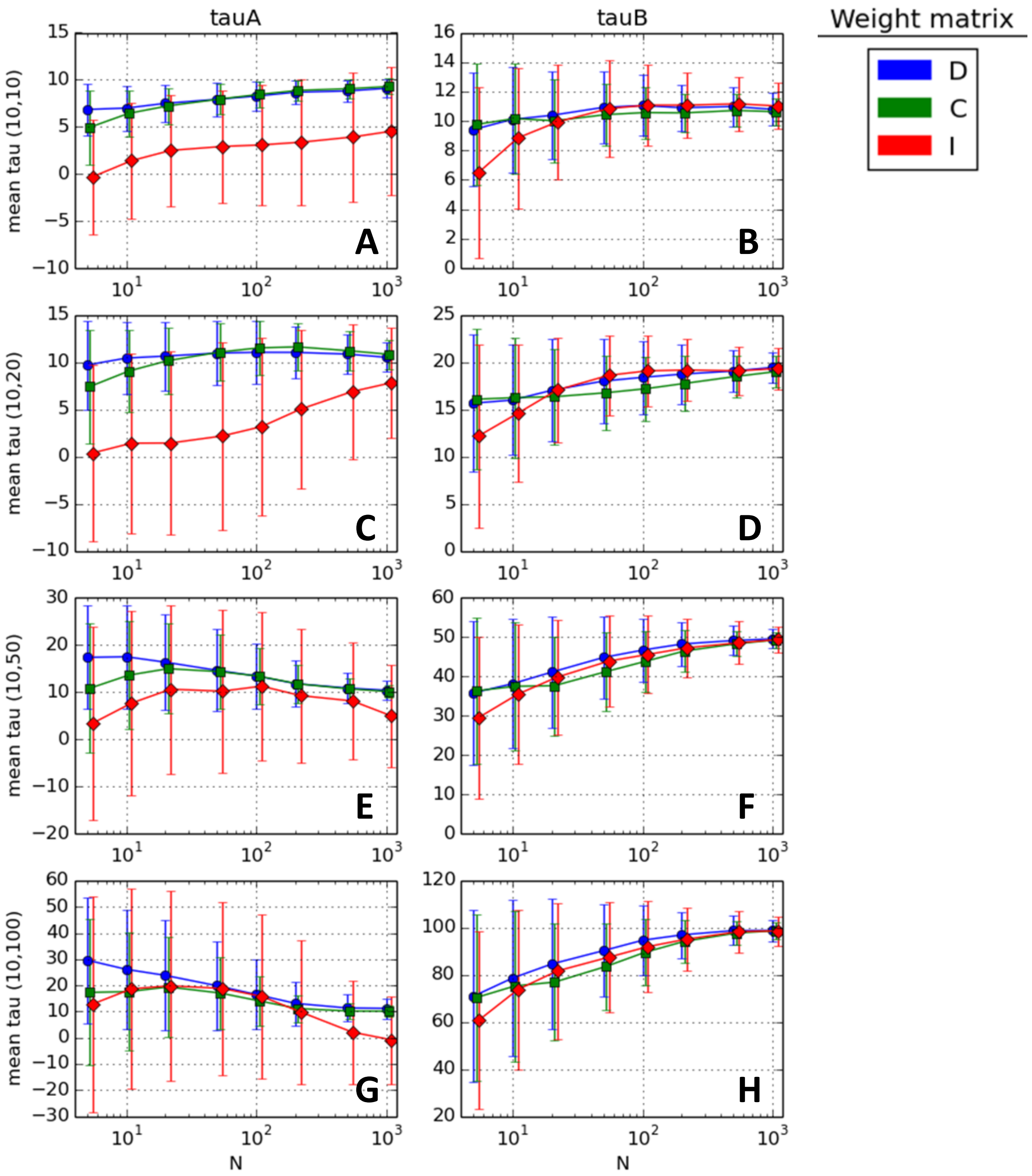

FIGURE S5. Results for third-order cost function.

S6. Fourth-order cumulant cost function comparison

The complete results for the fourth order cost function (see Materials and Methods for full description) as applied to a two-step reaction mechanism are shown in Figure S6. For the smaller estimates (A, C, E, G), the bias generally decreases as N increases. While the diagonal jackknife weight matrix and the full jackknife matrix generally have comparable bias, the identity matrix is consistently more biased. Additionally, for the smaller estimate when the two residence times are equal (A), estimates converge to a value less than the true parameter (9 vs. 10 s). For the larger estimates (B, D, E, F), the bias generally decreases as N increases, with the diagonal jackknife, full jackknife, and identity matrix in order of least to greatest bias. Additionally, for the larger estimate under these conditions (B), estimates converge to a value greater than the true parameter (11 vs. 10 s).

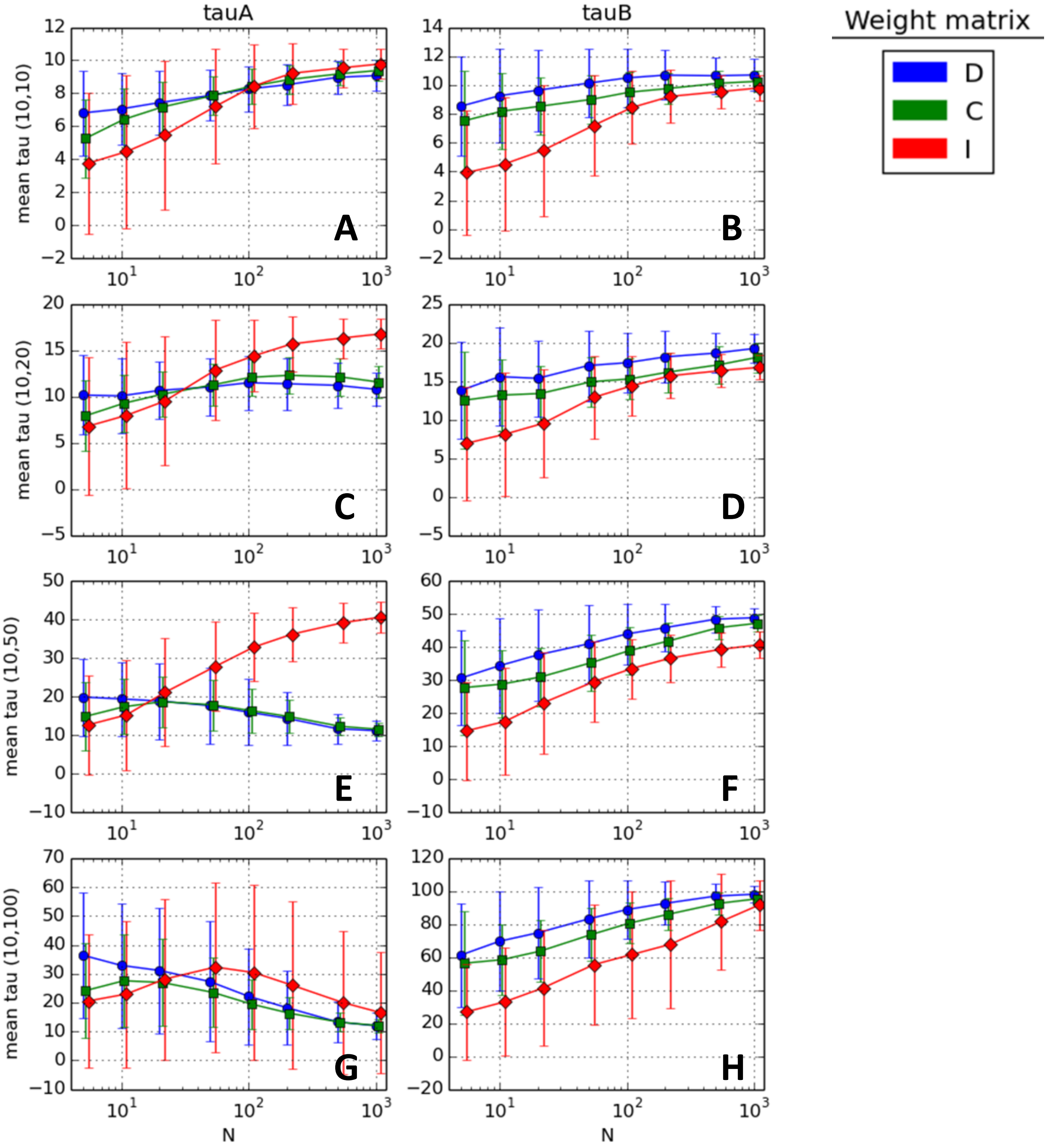

FIGURE S6. Results for fourth-order cost function.

S7. Second pass GMM comparison

The complete results for the GMM second pass (third and fourth order) compared to the GMM first pass (second order) as applied to a two-step reaction mechanism (see Materials and Methods for full description) are shown in Figure S7. D-matrix weight was used (interpolated D on the second pass). For all estimates, the bias generally decreases as N increases. At high N, the addition of a second pass of the GMM does not reduce bias. At low N for smaller estimates when residence parameters are closer in value (A, B), the second pass reduces bias slightly. At low N for smaller estimates when residence parameters are further in value (E, G), the second pass increases bias slightly. At low N for larger estimates when residence parameters are equal (B), the second pass increases bias slightly. At low N for larger estimates when residence parameters are closer in value (D), the second pass reduces bias slightly. All three conditions are comparable for larger estimates when residence parameters are further in value (F, H). For the smaller estimate when the two residence times are equal (A), estimates converge to a value less than the true parameter (9 vs. 10 s), while for the larger estimate under these conditions (B), estimates converge to a value greater than the true parameter (11 vs. 10 s).

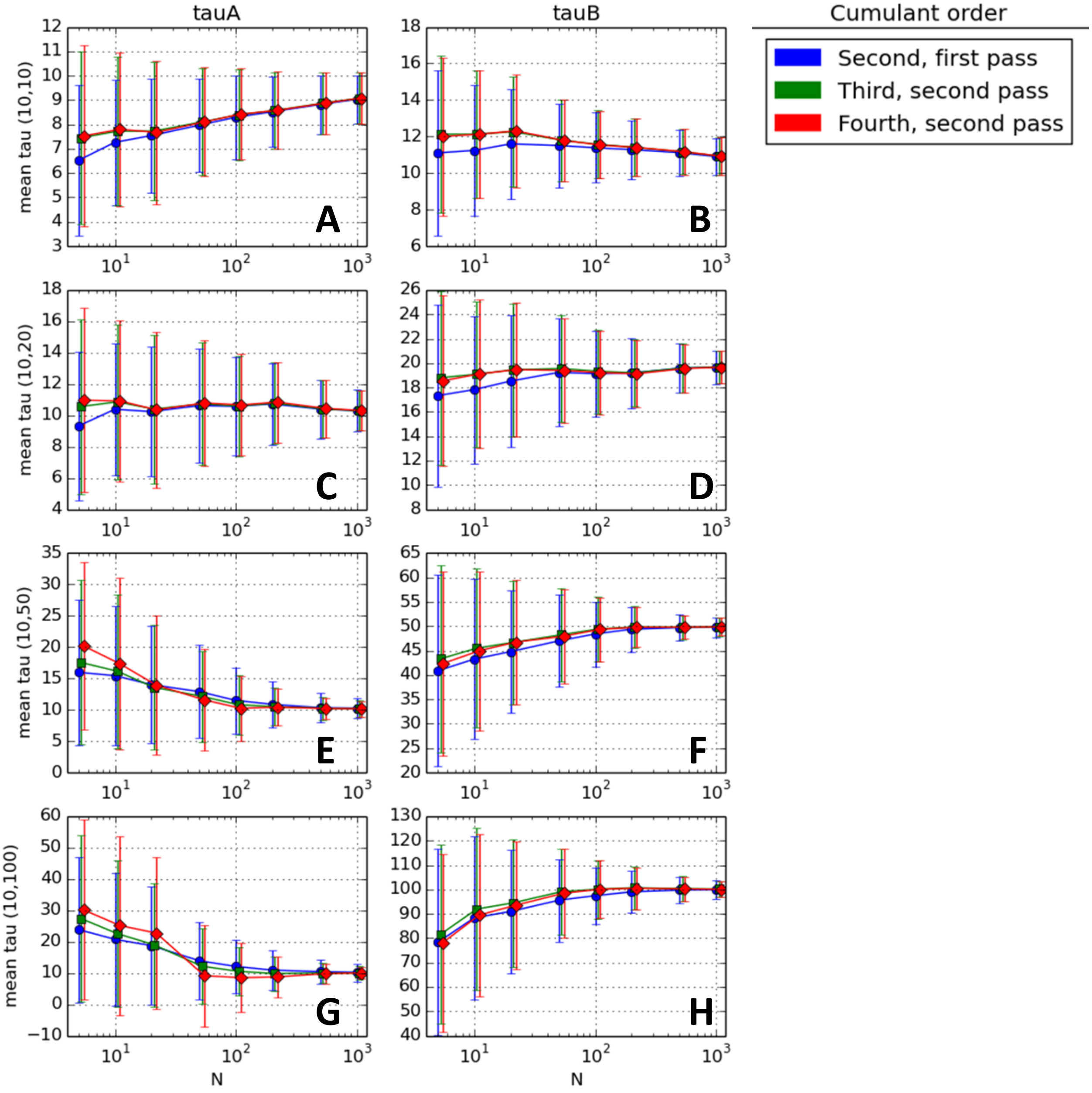

FIGURE S7. Results for second pass of the GMM.

S8. NL-LSQM comparison

The complete results for the NL-LSQM compared to a single-pass, second order, D-matrix GMM as applied to a two-step reaction mechanism (see Materials and Methods for full description) are shown in Figure S8. For all estimates, the bias generally decreases as N increases. For the smaller estimates (A, C, E, G), bias is comparable for least squares and GMM, with the exception of N < 20. In this case, the least squares gives large negative values with large variances which are not plotted here. For the larger estimates (B, D, F, H), bias difference between the GMM and least squares minimizes as the difference between the two residence times increases. As N decreases, bias worsens for least squares more dramatically than for the GMM. For the smaller estimate when the two residence times are equal (A), estimates converge to a value less than the true parameter (9 vs. 10 s), while for the larger estimate under these conditions (B), estimates converge to a value greater than the true parameter (11 vs. 10 s).

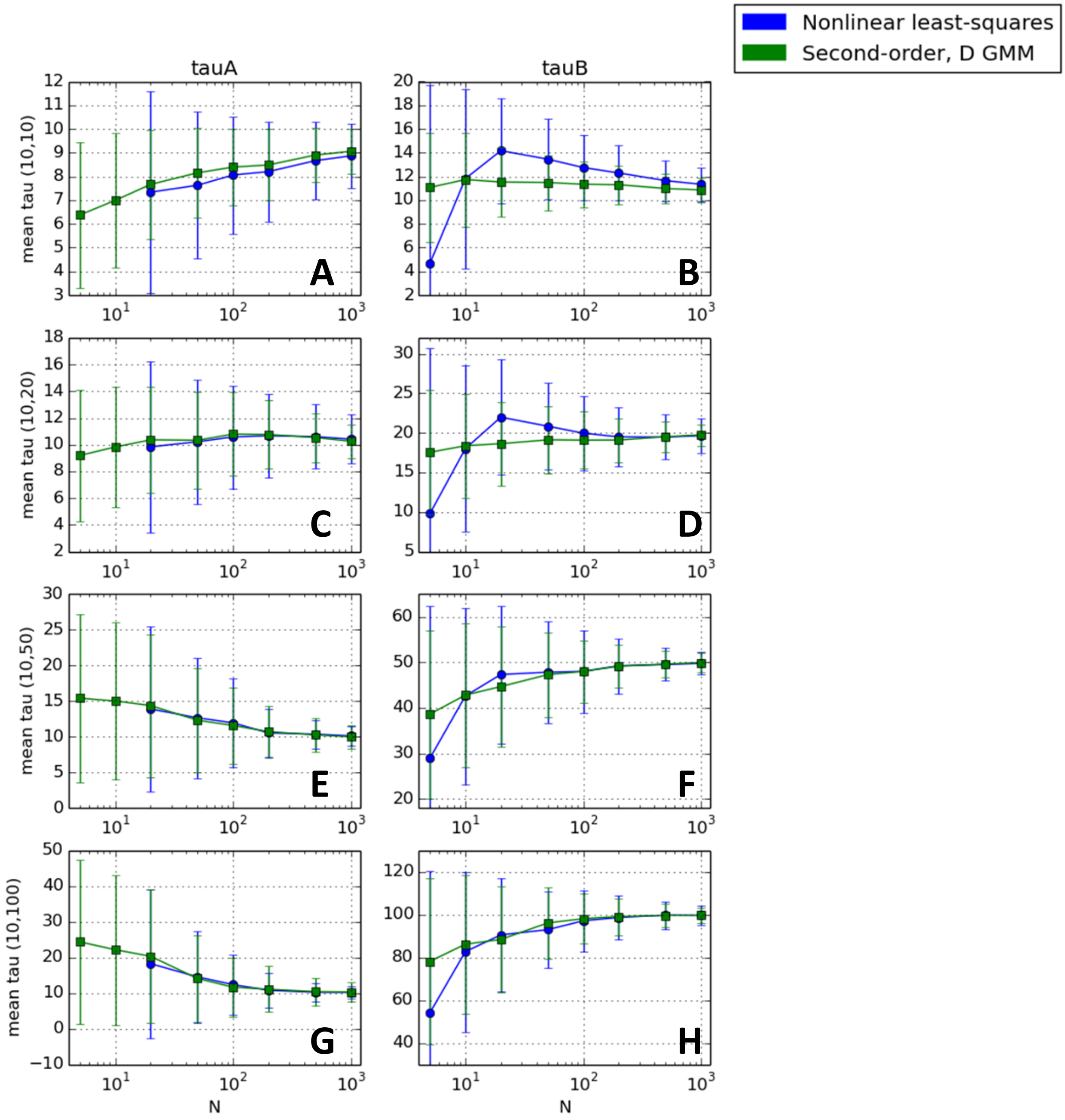

FIGURE S8. Results for comparison of nonlinear least-squares to single-pass, second-order, D-matrix GMM.

S9. Extended analysis for 3-step reaction mechanism with parameters (10s,10s,50s)

Following from Figure 5 in the main text, these results below show that the bias eventually reduces for all three estimates of individual dwell times for the set (10s, 10s, 50s). As observed previously with two identical dwell times, one converges to a negative bias and the other converges to a positive bias, both roughly 2-3 s off from the true value. Note that this is different than the two-step case, where values for identical dwell times converged to +/- 1 s, i.e., use of a three-step mechanism where two steps are very close in dwell time will produce greater bias than if there are only two total steps. Third order again generally performs best here, but this only occurs at high N (> 100) for the smallest dwell time. This GMM utilized the diagonal jackknife weight matrix.

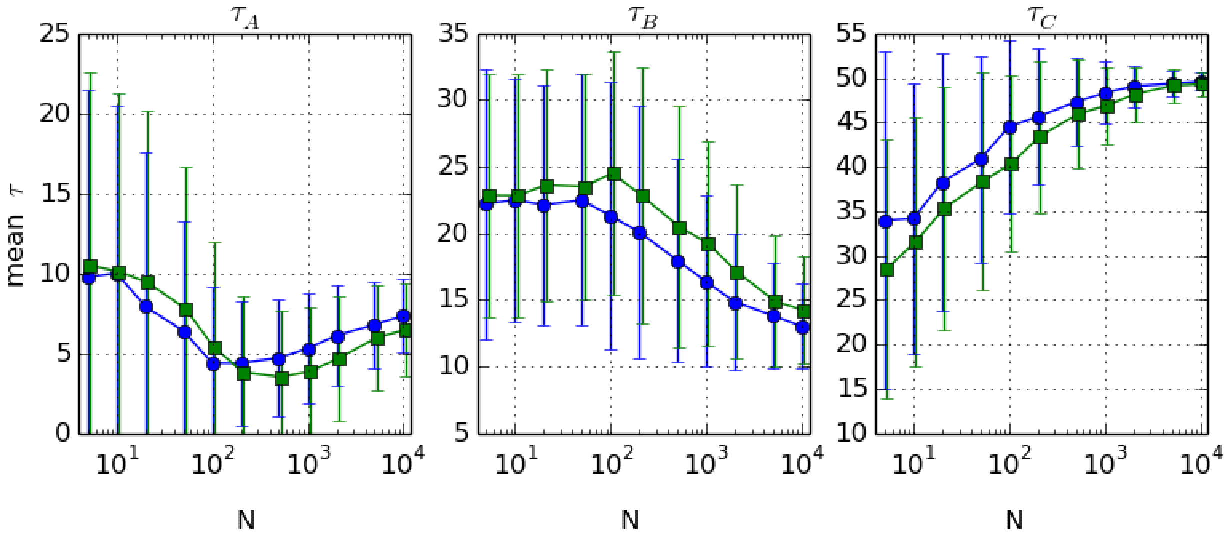

FIGURE S9. Extended results for testing GMM with a three-step mechanism. The panels ordered left to right show the values of the three returned parameters in increasing order. Blue circles are third order cumulant and green squares are fourth order cumulant. Diagonal jackknife weight matrix used. This figure extends the results of Figure 5D-F out to 10,000 samples.

S10. Analysis of 3-step reaction mechanisms with parameters (1s,10s,100s) and (1s, 20s, 400s)

These results below show that the "just specified," diagonal jackknife weight matrix for the sets (1s, 10s, 100s) and (1s, 20s, 400s) clearly distinguishes three separate dwell times. As expected, the value which is furthest away from the other two in value is estimated with the least bias (as expected from two-step cases where identical dwell times introduced more bias, as well as from the lower accuracy of identical dwell time pairs within the three-step in Figure 5). Estimates for the lower values become poorer as this difference becomes more drastic (since the two smaller dwell times become similar in magnitude compared to the larger one), but the GMM still returns values which reflect the true values relative to each other. This GMM utilized a third order diagonal jackknife weight matrix.

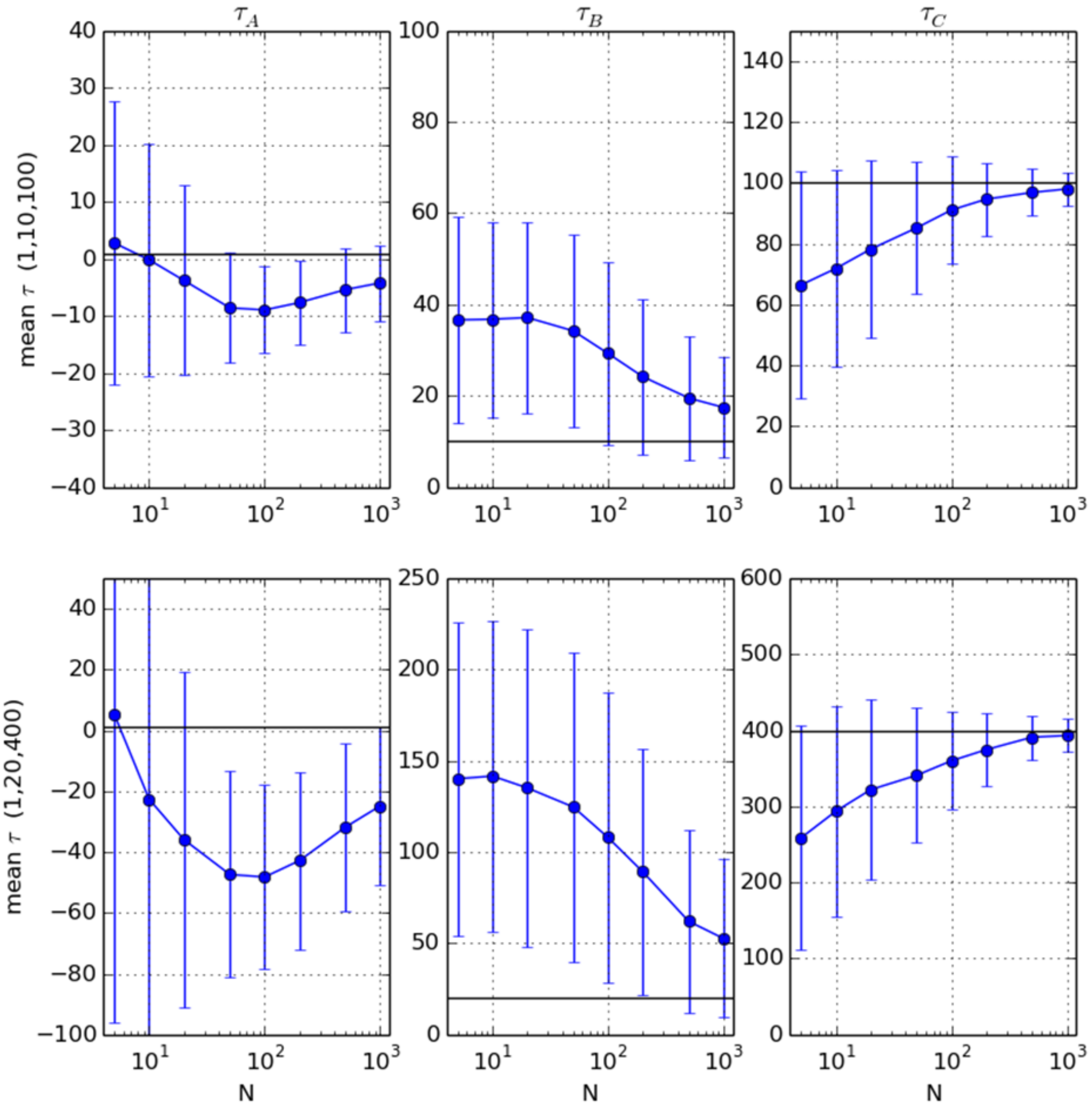

FIGURE S10. Results for testing third order GMM with a three-step mechanism containing large differences in the intrinsic decay rates. The panels ordered left to right show the values of the three returned parameters in increasing order. The solid black horizontal line indicates the value of the true decay time.

S11. Relation between 2nd order just-specified GMM and CMM

The purpose of this section is to clarify the relationship between the CMM and the GMM in the case of the 2nd order just-specified case. We will also prove the statement made in the Discussion section that when the CMM fails to return a real solution, the GMM will return a solution with two identical parameters (a double root).

We first consider the most general form of the cost function for a bi-exponential distribution (two step mechanism) in the 2nd order GMM.

$$Q(\tau_1, \tau_2, m, v) = A(\tau_1 + \tau_2 - m)^2 + 2B(\tau_1 + \tau_2 - m)(\tau_1^2 + \tau_2^2 - v) + C(\tau_1^2 + \tau_2^2 - v)^2 \;. \quad \text{(S1)}$$

In this expression, we have written the cost function, Q, as a function of the two parameters, τ_1 and τ_2, and the sample mean m, and variance v, as determined from the experimental data. The constants A, B, and C are the coefficients from the positive definite weight matrix. The fact that this matrix is positive definite guarantees that the function Q will have positive values for all values of the moment functions (the terms in parentheses) unless the moment functions are all identically zero.

In general, the cost function is a quartic polynomial in τ_1 and τ_2. However, the special form of the function can be made clear by using the following coordinate transform. First, since the cost function is symmetric under the interchange of τ_1 and τ_2, we can limit our consideration to the lower half (τ_1,τ_2) plane defined by $\tau_1 > \tau_2$. We then define the mapping of this half plane into the (ρ, s) plane by the following coordinate transformation.

$$s = \tau_1 + \tau_2, \qquad -\infty \leq s \leq \infty \qquad \text{(S2)}$$

$$\rho = \tau_1^2 + \tau_2^2, \qquad \rho \geq \frac{1}{2}s^2 \qquad \text{(S3)}$$

This mapping is illustrated in Fig S11, where we can see that the half plane region defined by $\tau_1 > \tau_2$ is mapped into the central (unshaded) region defined by the parabola $\rho = ½ s^2$. Points in the interior of the $\tau_1 > \tau_2$ half plane in the (τ_1,τ_2) plane are mapped into the interior of the parabola, and points on the line $\tau_1 = \tau_2$ are mapped onto the parabola.

The form that the cost function takes in these new coordinates is a simple paraboloid,

$$Q'(s, \rho, m, v) = A(s - m)^2 + 2B(s - m)(\rho - v) + C(\rho - v)^2. \qquad \text{(S3)}$$

The solution to the GMM is then equivalent to the minimization of Q' over (ρ, s) within the region bounded by the parabola shown in Fig S11. To see how this clarifies the behavior of the minima of the cost function Q, we consider the extension of Q' to the entire (ρ, s) plane. This function has a single global minimum located at its zero, $(\rho, s) = (v, m)$. When the zero lies inside the parabola in the (ρ, s) plane, this zero maps onto a zero (and hence global

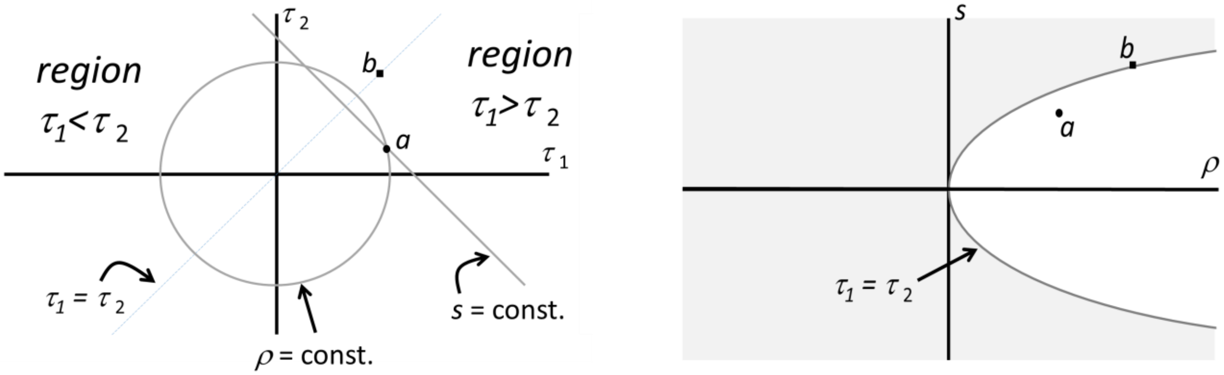

FIGURE S11. Illustration of the coordinate transformation of parameter space. The left panel shows the (τ_1,τ_2) plane and curves of constant s and ρ. The right panel shows the mapping of the region the $\tau_1 > \tau_2$ into the (ρ, s) plane.

minimum) of the original cost function $Q(\tau_1,\tau_2)$ in the $\tau_1 > \tau_2$ half plane. By reflection, there is another zero in the $\tau_1 < \tau_2$ half plane, and therefore two and only two minima, each at one of only two zeros of the cost function. Since the weight matrix is positive definite, this implies the moment conditions are specified exactly, and therefore these minima are also solutions to the CMM.

When the unique zero of $Q'(\rho, s)$ lies outside the parabola (that is, in the shaded region in Fig S11), the minimum lies on the parabolic boundary to the region. This point maps onto one and only one point in the (τ_1,τ_2) plane, and therefore, there is one and only one minimum to the cost function $Q(\tau_1,\tau_2)$. In this case the GMM returns two identical roots. The minimum is not at a zero of $Q'(\rho, s)$, and hence, its exact value and location will depend on the values of the coefficients A, B, and C (the weight matrix of the GMM). It is clear that when the zero of the function lies on the bounding parabola, the GMM will also return two identical parameter values, however, these will be independent of the weighting coefficients.

The exact criteria for when the zero of the function $Q'(\rho, s)$ lies outside the bounding parabola is simply that the zero lies at a point where $v < \frac{1}{2} m^2$. Setting the moment functions present in Eq. S1 equal to zero and solving for the roots, we find that the roots are complex exactly when $v < \frac{1}{2} m^2$, which is the identical condition. Therefore, the CMM returns complex solutions exactly when the GMM returns identical parameters for the solution.

S12. Histograms of experimental data

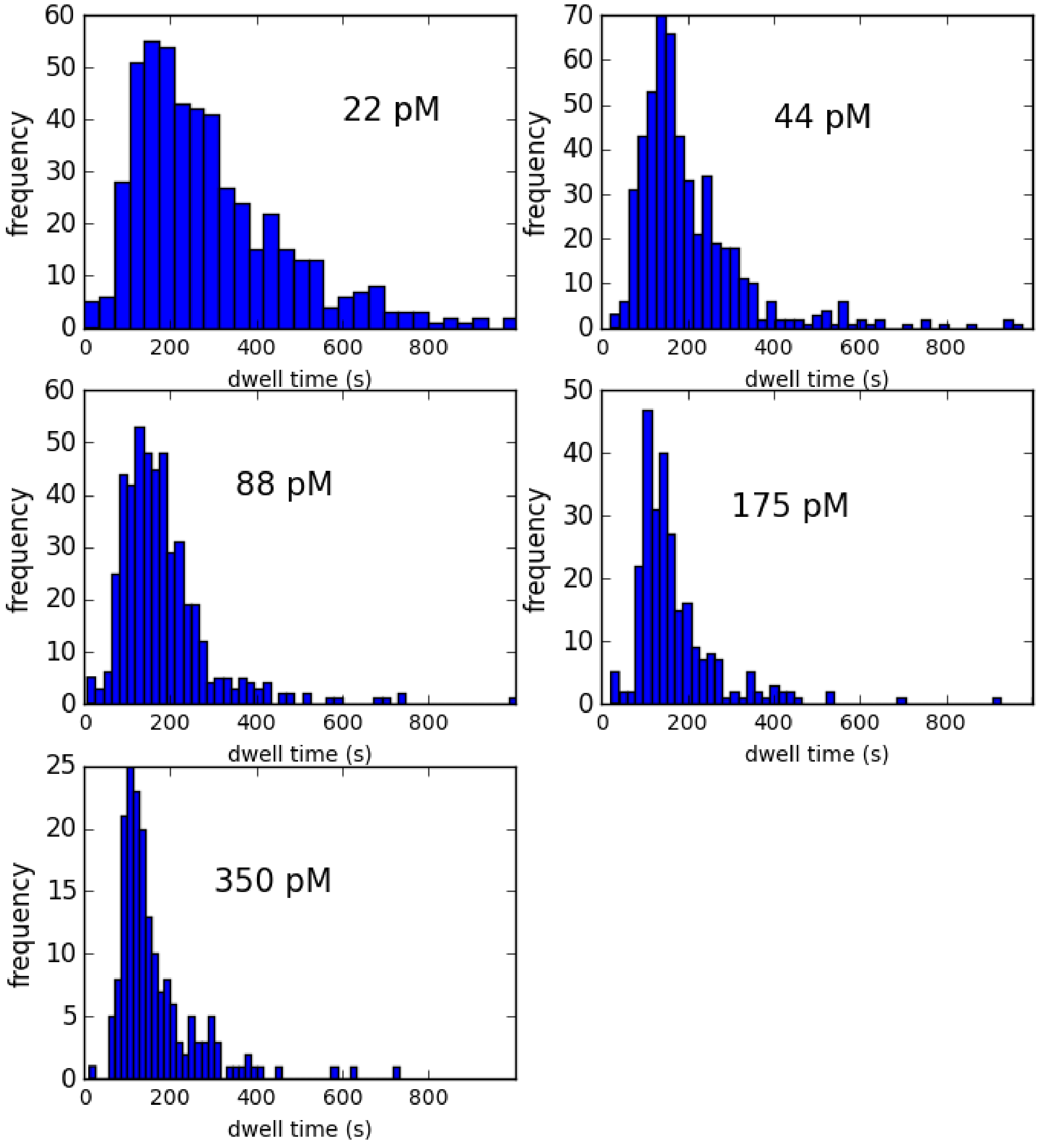

FIGURE S12. Histograms of all experimental data used in this work. Frequencies are number of counts in bin. The concentration of protein (NdeI) is shown in each panel. See Materials and Methods: Experimental data collection and processing for details.
